# Clinical Spectrum and Burden of Influenza-Associated Neurological Complications in Hospitalised Paediatric Patients

**DOI:** 10.3389/fped.2021.752816

**Published:** 2022-01-20

**Authors:** Michael Kwan Leung Yu, Cherry Pui Pik Leung, Wilfred Hing Sang Wong, Alvin Chi Chung Ho, Annie Ting Gee Chiu, Helen Hui Zhi, Godfrey Chi Fung Chan, Sophelia Hoi Shan Chan

**Affiliations:** ^1^Department of Paediatrics and Adolescent Medicine, Li Ka Shing Faculty of Medicine, The University of Hong Kong, Pokfulam, Hong Kong SAR, China; ^2^Department of Paediatrics and Adolescent Medicine, Queen Mary Hospital, Pokfulam, Hong Kong SAR, China; ^3^School of Public Health, Li Ka Shing Faculty of Medicine, The University of Hong Kong, Pokfulam, Hong Kong SAR, China

**Keywords:** influenza, cross-sectional study, paediatrics, neurological complications, encephalopathy

## Abstract

**Background:**

Influenza is one of the most common causes of acute respiratory tract infections around the world. Influenza viruses can cause seasonal epidemics. There remains limited information on the impact of both seasonal influenza A and influenza B related hospitalisations from neurological complications in paediatric populations in Asia.

**Objectives:**

To examine both the clinical spectrum and healthcare burden of influenza-associated neurological complications (IANCs) within the paediatric population of Hong Kong.

**Methods:**

We conducted a population-based retrospective study to identify all paediatric patients (<18 years) admitted to a public hospital in Hong Kong with a confirmed influenza A or B infection between 2014 and 2018 using the Clinical Data Analysis and Reporting System of the Hospital Authority. The clinical spectrum of the paediatric patients with IANCs was studied. The clinical burden of paediatric influenza patients with IANCs were compared to paediatric influenza patients without neurological complications.

**Results:**

A total of 28,016 children admitted to the paediatric wards diagnosed to have influenza A or B infection were identified, accounting for 5.7% (28,016/489,955) of total paediatric admissions. 67.3% had influenza A and 32.7% had influenza B, and 8.9% had IANCs. The mean annual incidence of IANCs in children was 57 per 100,000 population. The spectrum of IANCs in our paediatric patients included febrile seizures (80.6%), myositis (11.4%), seizures with fever (5.4%), influenza-associated encephalitis/encephalopathy (IAE) (2.6%) and rarely Guillain–Barré syndrome (0.04%). Most paediatric patients with IANCs (85.5%) presented at a young age of <6 years. Paediatric patients with IANCs had significant longer hospital stays (*p* < 0.001), higher percentages of mechanical ventilation use (*p* < 0.05) and PICU admissions (*p* < 0.001), and higher mortality rates (*p* < 0.001) compared to those without neurological complications. Amongst those with IANCs, IAE was the sole cause of all seven reported mortalities.

**Conclusions:**

Seasonal influenza A & B is a common cause of hospitalisation for paediatric patients in Hong Kong. We found neurological complications from influenza A and B caused a significantly higher clinical burden compared to those without neurological complications. Children in younger age groups (<6 years old) are at highest risk and thus increasing vaccination coverage to this age group is recommended.

## Introduction

Influenza is one of the most significant causes of acute respiratory diseases and has an annual incidence rate of 1–4 per 100,000 population (1, 2). Symptoms associated with influenza are very diverse, ranging from mild infections confined to the upper respiratory tract, such as cough and runny nose, to lethal conditions like pneumonia. Influenza can also cause a wide range of non-respiratory complications such as cardiac, neurological, and hematologic complications (3). Influenza can be differentiated as seasonal and pandemic. Seasonal influenza refers to the occurrence of existing influenza subtypes, while pandemic influenza refers to the emergence and global spread of novel strains of influenza A, resulting from the antigenic shift (4). Pandemic influenza is often associated with a higher severity of infection and increased mortality.

Following the 2009 H1N1 pandemic, interest in influenza-associated neurological complications (IANCs) surged. Different research groups have estimated that 6–19% of children with influenza infections were admitted for IANCs due to H1N1 (2, 5–7). The spectrum of IANC includes acute disseminated encephalomyelitis (ADEM), Reye's syndrome, Guillain-Barré syndrome (GBS), influenza-associated encephalitis/encephalopathy (IAE), and febrile seizures (8–10). Amongst these neurological complications, febrile seizures and IAE have been the primary foci of previous studies due to their higher incidence and severity respectively (11–13).

However, there are still limited population-based studies on the clinical characteristics and burden of seasonal IANCs in the paediatric age group (14). Therefore, we performed a retrospective population-based study on children <18 years old with influenza A or B infections who were admitted to public hospitals under the Hospital Authority (HA) of the Hong Kong Special Administrative Region (HKSAR) between 2014 and 2018. We calculated the incidence rate of IANCs in Hong Kong's paediatric population and compared the clinical burden of those with neurological complications to those without.

## Materials and Methods

### Data Collection *via* Clinical Data Analysis and Reporting System (CDARS)

CDARS is an electronic platform of the HA in which patients' clinical diagnoses, drug prescription details, laboratory investigation results, medical interventions, and length of hospital stay can be retrieved in an anonymous manner (15). Public hospitals under the HA manage 28,929 in-patient beds, which account for 85% of all in-patient admissions of HKSAR (16). We systematically collected the influenza hospitalisation, neurological symptoms and outcome of paediatric patients (<18 years) admitted to the paediatric departments of the 13 public hospitals in Hong Kong between January 2014 and December 2018.

### Admission and Hospitalisation of Paediatric Patients With Influenza A or B

Influenza cases were defined by a positive result confirming influenza A or influenza B by either influenza immunofluorescence assay or reverse transcription real time polymerase chain reaction of nasal pharyngeal aspirates. The proportion of influenza hospitalisations with respect to overall paediatric admission during the study period was also calculated.

### Influenza Associated Neurological Complications

The IANCs included in this study:

(1) Influenza-associated encephalopathy/encephalitis (IAE): alterations in consciousness including behavioural changes and evidence of central nervous system inflammation using cerebrospinal fluid/ magnetic resonance imaging (MRI) data. Acute necrotizing encephalopathy is characterised by the multiple bilateral brain lesions. MRI scan results was examined to find out any restricted diffusion in the T1 and T2 region.
(2) Benign febrile seizures: patients with non-focal seizures with fever aged between 6 months and <6 years old, with normal prior development, no known history of epilepsy, and other causes of seizure excluded.
(3) Seizure with fever: patients aged <6 months and ≥6 years old who develop seizures in association with fever.
(4) Myositis: inflammation of skeletal muscles with acute onset of muscle pain and weakness and raised creatine kinases levels.
(5) Guillain-Barré syndrome (GBS): acquired immune-mediated polyneuropathy with an acute onset of ascending weakness and rapid deterioration.

Influenza patients with any of the above conditions with an onset of neurological symptoms within 7 days of infection were defined as having IANCs. We then identified these patients via retrieval of the respective diagnostic codes from CDARS (Supplementary Table 1).

Data including the age, sex, type of influenza infection, length of stay, intensive care admissions, diagnoses, neurological complications, use of mechanical ventilation, presence of coinfection with bacteria or other viruses, uses of antiviral therapy, presence of pre-existing neurological or neurodevelopmental disorder and survival outcome of each patient was systematically collected from CDARS (Supplementary Table 2). The percentage of neurological complications and the annual incidence rate of influenza, as well as of IANCs and IAEs among different influenza subtypes and age groups (0–<2, 2–<6, 6–<12, and 12–<18 years) were calculated using age-specific population data provided by the Hong Kong Census and Statistics Department (Supplementary Table 3). The incidence rate of hospitalisation was weighted by 0.85 to reflect the proportion of patients served by public hospitals in Hong Kong (Supplementary Table 4).

To examine the clinical burden of IANCs, we compared the in-patient length of stay, need for PICU admission, use of mechanical ventilation, and the mortality rate, between influenza patients with neurological complications and those without. The vaccination coverage rate of seasonal influenza during the study period was extracted from the Centre for Health Protection. This study was approved by the Institutional Review Board of the University of Hong Kong (UW19-308).

### Statistics

Data analysis was performed using the Statistical Package for Social Sciences (IBS SPSS Statistics 25 Inc., Somers, NY). Length of stay of patients with IANCs and influenza patients without neurological complications were compared using the unpaired *t*-test. PICU admission, mechanical ventilator use, and death rates were compared using Chi-square tests as appropriate. A 95% confidence interval was calculated for the incidence rate. All analyses were two-tailed. The level of significance was set at *p* ≤ 0.05.

## Results

### Admission of Seasonal Influenza in Hong Kong From 2014 to 2018

A total of 28,016 admissions due to seasonal influenza A or B amongst patients aged <18 years of age were identified from CDARS within the 5-year study period (2014–2018), accounting for 5.7% (28,016/489,955) of total paediatric admissions. Amongst these patients, 67.3% (18,841) had influenza A and 32.7% (9,175) had influenza B. Just over half of these patients (53.9%; 15,100) were male. The median age for hospitalisation due to influenza was 3.3 years for influenza A and 5.0 years for influenza B (Table 1).

**Table 1:** Characteristics of paediatric patients admitted with influenza infection and influenza-associated neurological complications (IANCs) between 2014 and 2018.

**Year**	**2014**		**2015**		**2016**		**2017**		**2018**		**2014–2018**		
**Paediatric admission**	**91,973**		**90,349**		**102,740**		**104,376**		**100,517**		**489,955**		
**Types of Influenza**	**A**	**B**	**A**	**B**	**A**	**B**	**A**	**B**	**A**	**B**	**A**	**B**	**A+B**
**Influenza admission**	**2,628**	**1,353**	**3,055**	**686**	**4,017**	**2,368**	**5,721**	**955**	**3,420**	**3,813**	**18,841**	**9,175**	**28,016**
Median age of Influenza admission (years, IQR)	3.1 (1.7–5.3)	5.2 (2.8–7.9)	3.2 (1.6–6.4)	5.2 (2.7–7.5)	3.2 (1.6–5.0)	5.1 (3.0–7.9)	3.5 (1.8–6.0)	5.5 (3.2–8.2)	3.7 (2.0–5.9)	4.7 (2.5–7.4)	3.3 (1.7–5.7)	5.0 (2.7–7.7)	3.8 (2.0–6.5)
Male (%)	1,429 (54.4)	700 (51.7)	1,686 (55.2)	382 (55.7)	2,212 (55.1)	1,251 (52.8)	3,105 (54.3)	498 (52.1)	1,840 (53.8)	1,997 (52.4)	10,272 (54.5)	4,828 (52.6)	15,100 (53.9)
**Patients with IANC**	
Overall IANC^a^ (%)	332 (12.6)	126 (9.3)	333 (10.9)	52 (7.6)	423 (10.5)	191 (8.1)	465 (8.1)	77 (8.1)	258 (7.5)	226 (5.9)	1,811 (9.6)	672 (7.3)	2,483 (8.9)
Febrile Seizure (%)	306 (92.2)	83 (65.9)	292 (87.7)	32 (61.5)	374 (88.4)	121 (63.4)	404 (86.9)	40 (51.9)	202 (78.3)	147+(65.0)	1,578 (87.1)	423 (62.9)	2,001 (80.6)
Myositis (%)	7 (2.1)	26 (20.6)	15 (4.5)	11 (21.2)	25 (5.9)	51 (26.7)	24 (5.2)	32 (41.6)	33 (12.8)	60 (26.5)	104 (5.7)	180 (26.8)	284 (11.4)
Seizure with fever (%)	14 (4.2)	13 (10.3)	18 (5.4)	6 (11.5)	17 (4.0)	14 (7.3)	19 (4.1)	3 (3.9)	17 (6.6)	12 (5.3)	85 (4.7)	48 (7.1)	133+(5.4)
IAE (%)	5 (1.5)	4 (3.2)	8 (2.4)	3 (5.8)	7 (1.7)	5 (2.6)	17 (3.7)	2 (2.6)	6 (2.3)	7 (3.1)	43 (2.4)	21 (3.1)	64 (2.6)
<6 years old (%)	313 (94.3)	88 (69.8)	300 (90.1)	36 (69.2)	388 (91.7)	138 (72.3)	428 (92.0)	49 (63.6)	219 (84.9)	163 (72.1)	1,648 (91.0)	474 (70.5)	2,122 (85.5)
Male (%)	217 (65.4)	79 (62.7)	222 (66.7)	36 (69.2)	252 (59.6)	132 (69.1)	306 (65.8)	51 (66.2)	171 (66.3)	145 (64.2)	1,168 (64.5)	443 (65.9)	1,611 (64.9)

a *2.5% (61/2,483) of cases carried pre-existing neurological disease; 1.1% (27/2,483) of cases had co-infection with other virus and bacteria*.

*The data are presented as number of patients (%). The identified influenza-associated neurological complications (IANCs) included febrile seizures, myositis, seizure with fever and influenza-associated encephalitis/encephalopathy (IAE). The only patient with Guillain-Barré syndrome after influenza A infection in 2017 was not included in this table*.

### Influenza-Associated Neurological Complications

Two thousand four hundred eighty-three patients with IANCs were identified, giving an overall IANC incidence rate of 8.9% (Table 1). 85.5% of patients with IANCs occurred in children <6 years of age. The median age of patients with IANCs was 3.0 years. Two-thirds of the patients with IANCs were male (64.9%) (Table 1). The most common IANC was febrile seizures, which accounted for 80.6% of all IANC cases, followed by myositis (11.4%) and seizures with fever (5.4%). 2.6% (64/2,483) of patients had IAE. One patient with influenza A had GBS (Table 1).

The mean annual incidence of IANCs among children <18 years of age was 57 per 100,000 population (Supplementary Table 3). Between the different age groups, the mean annual incidence was highest in children aged <2 years (149 per 100,000 population) and lowest in children aged 12– <18 years (2 per 100,000 population). The overall mean incidence of IANCs was significantly higher in influenza A at 42 per 100,000 children compared to 15 per 100,000 children in influenza B (Supplementary Table 3). The mean annual incidence of IAE for all children (<18 years old) was 14.8 per 1,000,000 population and was highest in children aged <2 years (22.4 per 1,000,000 population).

### Trend of IANCs During 2014–2018

**Figure 1:**
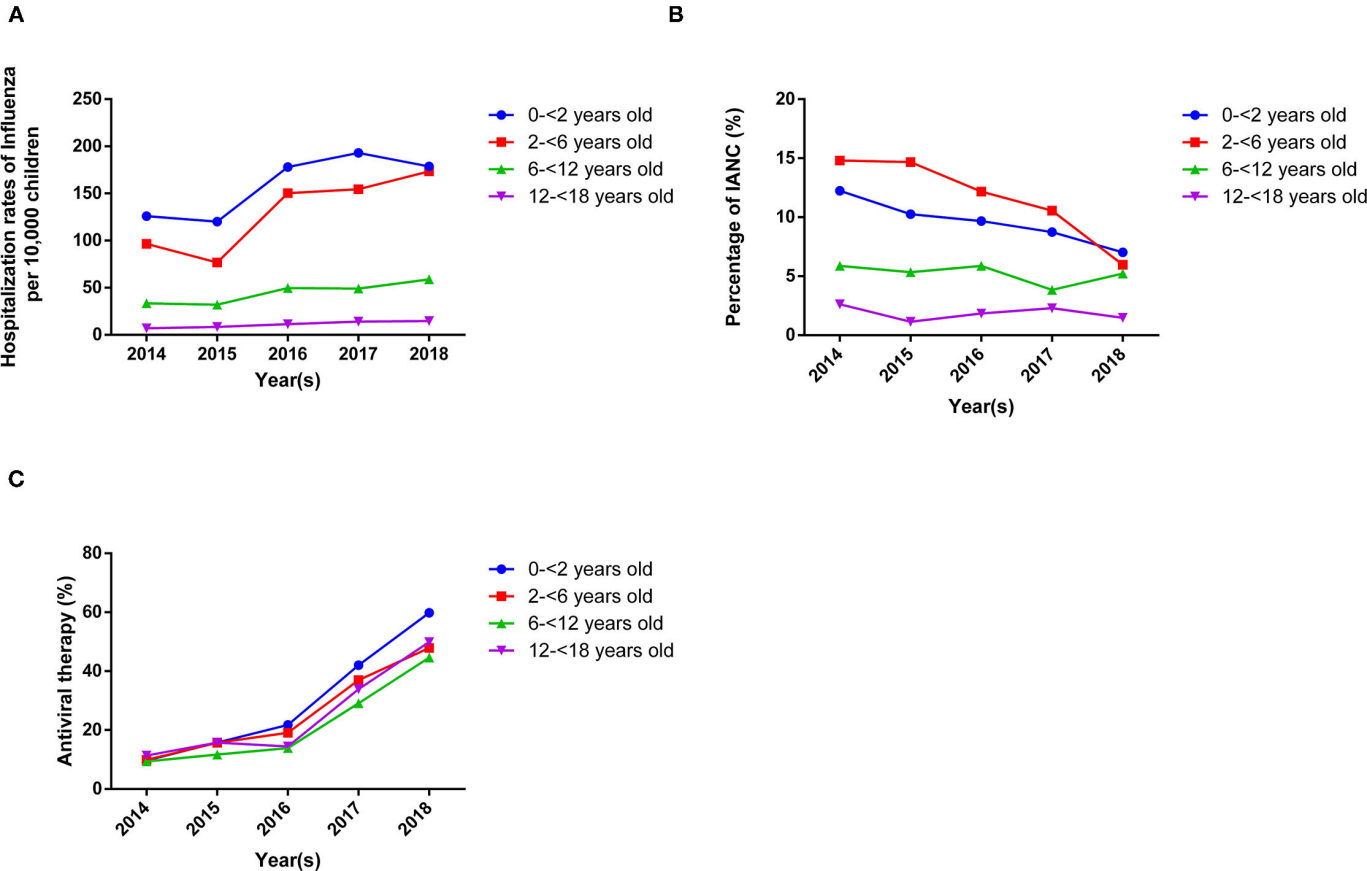
The hospitalisation rate, the percentage of IANCs, and the use of anti-viral therapy in paediatric in-patients diagnosed to have influenza A or B infection during the study period 2014 to 2018. **(A)**. The hospitalisation rate of paediatric patients with influenza A or B increased from 2014 to 2018 for all age groups. **(B)** The percentage of total IANCs in the admitted paediatric patients (<18 years old) with influenza A or B infection decreased from 2014 to 2018 for all age groups. **(C)** The percentage of anti-viral therapy (oseltamivir) in admitted paediatric patients (<18 years old) with influenza A or B infection increased from 2014 to 2018 for all age groups.

Although the influenza hospitalisation rate increased from 46 to 83 per 10,000 children (<18 years old) from 2014 to 2018 (Figure 1A), annual IANC incidence amongst paediatric in-patients with influenza A or B decreased from 11.5% in 2014 to 6.7% in 2018 (Table 1). This decline in the annual IANC percentage was observed in all paediatric age groups (Figure 1B).

### Clinical Spectrum of IANCs

Benign febrile seizures were the most common neurological complication in both influenza A and influenza B (Table 1). Overall, patients with influenza A had a higher percentage of febrile seizures compared to influenza B (8.4 vs. 4.6%, *p* < 0.001). The percentages of seizure with fever and IAE were similar between influenza A and influenza B patients (0.5 vs. 0.5%; 0.2 vs. 0.2%, *p* > 0.05). On the other hand, patients with influenza B showed a higher percentage of myositis than influenza A (2.0 vs. 0.6%, *p* < 0.001) (Supplementary Figure 1A). In the younger age groups (0– <2 years old & 2– <6 years old), the incidence of IANCs in influenza A patients was higher than that of influenza B (10.0 vs. 6.7%: 12.2 vs. 9.4%, *p* < 0.001). In the older age groups, however, influenza B patients showed a higher incidence of IANCs compared to influenza A (6.1 vs. 4.3% in 6– <12 years old, *p* < 0.01; 1.6 vs. 2.1 % in 12– <18 years old, *p* > 0.05) (Supplementary Figure 1B).

### Clinical Characteristics and Burden of IANCs

Paediatric patients with IANCs had significant longer hospital stays (*p* < 0.001), higher percentages of mechanical ventilation use (*p* < 0.05) and PICU admissions (*p* < 0.001), and higher mortality rates (*p* < 0.001) compared to those without neurological complications (Table 2).

**Table 2:** Comparison of the clinical characteristics and burden of hospitalised influenza admission with neurological complications (IANCs) and those without neurological complications (Non-IANC) between 2014 and 2018.

	**Non-IANC (*N* = 25,533)**	**IANC (*N* = 2,483)**	***p*-value**
Pre-existing neurological or neurodevelopmental disorder *N* (%)	562 (2.20)	61 (2.46)	0.410^e^
Length of stay (Days)
Mean (SD)	2.56 (3.21)	2.93 (3.45)	<0.001^f^
Use of antiviral therapy *N*^a^ (%)	7,238 (28.3)	917 (36.9)	<0.001^e^
Mechanical ventilation *N* (%)	65 (0.25)	12 (0.49)	0.036^e^
PICU admission *N* (%)	169 (0.66)	65^b^ (2.63)	<0.001^e^
Deaths *N* (%)	3^c^ (0.01)	7^d^ (0.28)	<0.001^e^

a *99.98% (8,154/8,156) of antiviral therapy referred as Oseltamivir. One patient used Peramivir and another patient used Zanamivir*.

b *Two-thirds (114/169) of the influenza patients admitted to PICU were admitted due to respiratory manifestations, including respiratory distress or failure resulted resulting from influenza pneumonia with/ or without other bacterial or viral co-infections, or severe croup, or asthma exacerbation. Other less common reasons for PICU admission included myocarditis, septicaemia, and exacerbation of underlying diseases such as deterioration of diabetes mellitus control*.

c *One patient died of pneumonia and septicaemia with pseudomonas and influenza A co-infection; one patient died of pneumonia and septicaemia with staphylococcus and influenza A co-infection; on patient with influenza B infection died of myocarditis and severe heart failure*.

d *All 7 IANC-related deaths were caused by IAE*.

e *Chi-square test*.

f *Non-paired t-test PICU, Paediatric Intensive Care Unit*.

Overall, IAE had the greatest clinical burden among IANC group. Patients with IAE had the highest median length of stay (6 days) compared to the other IANCs (2 days). IAE also accounted for 75% (9/12) of mechanical ventilation use and 63% (41/65) of PICU admission within the IANC group. Amongst those with IANCs, IAE was the sole cause of all seven reported mortalities. The mortality rate of IAE was 10.9% (7/64), and all deaths occurred amongst those <7 years old.

Other reasons of PICU admission for IANC patients included respiratory distress/respiratory failure (29%; 19/65), status epilepticus (SE) either from the benign febrile seizures or seizures with fever groups (6%; 4/65) and GBS (1/65).

Increase use of antiviral therapy (oseltamivir) was observed in all paediatric age group (Figure 1C). The use of antiviral therapy (osteltamivir) in patients with neurological complications was also found higher than those without neurological complications (36.9 vs. 28.3%, *p* < 0.001) (Table 2). Eighty percent (51/64) of the IAE patients were treated with antiviral therapy.

Only 2.46% (61/2,483) of paediatric influenza patients with neurological complications carried a pre-existing neurological or neurodevelopmental disorder. These include epilepsy, autism, and developmental delays.

## Discussion

Our study found seasonal influenza was one of the major causes for paediatric hospitalisation in the 5-years study period (2014 to 2018), generating a significant clinical burden on the Hong Kong healthcare system. Using data extracted from the CDARS system, we examined more than 28,000 seasonal influenza admissions of paediatric patients from 2014 to 2018. Of the few published studies investigating IANCs (1, 2, 5, 7, 13, 14, 17, 18), our study includes one of the largest numbers of paediatric patients with influenza A and B. The IANC percentages (8.9%) in our Hong Kong-based study are slightly higher but overall comparable to previous studies in Australia and the United States, which reported seasonal IANC percentages of 7.6 and 8.6%, respectively (1, 14). Importantly, 85.5% of the IANC cases were patients aged <6 years old. This supports the findings from another study from South Korea that reported a mean age of children hospitalised by influenza A-associated neurological complications of 5.9 years old (19). This may support the fact that younger patients are more susceptible to the complications of influenza infections.

### Clinical Spectrum of IANCs

We observed a spectrum of IANCs in the paediatric populations. The IANC that most commonly led to hospitalisation was febrile seizures (80.6% of IANCs). Only 1.1% of the febrile seizure group required PICU admission, and there was no associated mortality. As explained by Prerna et al. (20), febrile seizures are a major complication in influenza infections, however most patients recover well.

The proportion of febrile seizure cases admitted in our cohort was much higher compared to that of western countries. From a study in United States, only 37.5% (27/72) of patients with IANC admitted were due to febrile seizure (1). Additionally, another study from Australia showed that only 26% (14/54) of IANC are due to febrile seizure (14). This may be due to the difference in the clinical practise managing febrile seizure in our locality compared to western countries. According to the American Academy of Pediatrics, Royal College of Physicians, and the British Paediatric Association, simple febrile seizures that last <15 min and do not recur within 24 h usually do not require further evaluation in hospital (21, 22). In our study, the higher percentage of IANC admissions related to benign febrile seizures could reflect the parental anxiety, which could be attributed to the excessive media reports on the few annual influenza-related deaths (23). Moreover, when the children developed febrile seizures at home, many parents did not consult a family doctor and instead directly attended the emergency department. The lack of primary care physicians or family doctors in Hong Kong leads to the difficulty to set up the gatekeeping roles from primary care providers (24, 25). This may explain the higher percentage of IANC admissions related to febrile seizure in Hong Kong. Currently the Hong Kong College of Family Physicians is working closely with the government to increase the opportunity of family doctors' training. Further promotion of family medicine careers, as well as more public education on the benign nature and management of febrile seizures, would help to utilise medical resources more effectively and lower the healthcare burden of IANC-related febrile seizure admissions.

The second most common IANC leading to hospital admissions in our cohort was myositis (11.4%). We found that myositis was more prevalent in influenza B than influenza A infection, which confirms previous literature findings (26–28). We also found a single case of GBS after an influenza A infection. GBS is a rare neurologic complication and it is thus difficult to estimate the incidence rate (29). Similar to Glaser et al. (30), we could only find one case from this population-based cohort (*n* = 2,069).

Only a small percentage of patients with IANCs in our cohort carried pre-existing neurological diseases or neurodevelopmental disorders (2.5%), unlike other IANC studies in Italy and Taiwan (31, 32) (33 and 40%, respectively). The difference could be due to the high admission rate of benign febrile seizure in our studied population. We also recorded a low percentage (1.1%) of co-infection with other virus and bacteria in IANC cases. The pathogens included *S.pyogenes, H.influenzae* and Rotavirus which have been reported in previous studies (33, 34). No IAE patient in our cohort was found to have a co-infection.

### Significant Clinical Burden of IANCs

The patients with IANCs had longer in-patient length of stays, higher rates of antiviral therapy use and mechanical ventilator use, and higher PICU admissions and influenza-related deaths when compared to other influenza in-patients without neurological complications. Our data supports previous findings that influenza in-patients with neurological complications have statistically significantly higher ICU admissions compared to influenza patients without neurological complications (18). Patients with IAE had the longest in-patient hospital stay, highest rates of antiviral therapy use, PICU admissions and mechanical ventilator use, and was the only cause of mortality, making it the most severe IANC. All seven cases of IANC-related deaths occurred within 13 days of influenza admission. All deceased patients were below 7 years old (Range: 1.4–6.9 years old). The high mortality rate of 10.9% (7/64) in our patients with IAE echoes the poor clinical outcomes found in previous studies in Japan and Australia, with mortality rates of 7.8% (22/283) and 15% (2/13), respectively (13, 14). Apart from IAE, other IANC cases had good recovery with no mortality despite PICU admissions.

### Changes in Incidence of IANC Between 2014 and 2018

Between 2014 and 2018, we observed an increased admission rate of paediatric patients with influenza A or B infections. This could be related to the increased availability of influenza diagnostic tests in both private family doctor clinics and public hospitals, as well as increased public awareness of the neurological risks of influenza, especially following local mass media coverage of the death of a young child due to acute necrotising encephalopathy (ANE) associated with influenza infection in 2016. As such, it is possible that more parents preferred early admission and closer observation for their child following an influenza diagnosis especially when presented with convulsion, thus explaining the increased rate of admissions (35).

Although the admission rate increased, there was a decline in the percentage of IANCs amongst paediatric in-patients. This coincided with the promotion of universal influenza vaccinations in Hong Kong around the same period. In 2014/15, the government introduced the Childhood Influenza Vaccination Subsidy Scheme to subsidise seasonal influenza vaccination (SIV) in children aged 6 months to <6 years and expanded to children aged between 6 and 12 years in 2016/17 (36). This scheme boosted the vaccination coverage rate of children aged 6 months to 12 years from 17.4% in 2015/16 to 45.8% in 2018/19 (37, 38) (Supplementary Figure 2). Seasonal influenza vaccinations (SIV) are associated with lower symptom severity, decreased ICU admissions, and reduced mortality rates in infected individuals (39–41). According to a US-based paediatric study (39), full influenza vaccinations could reduce three-quarters of PICU admissions. Another study from Flannery et al. also echoes the effectiveness of influenza vaccines by showing that influenza vaccine had 65% efficacy in reducing paediatric influenza-associated deaths. Therefore, the increase in the SIV rate could be one of the reasons for the decreased incidence of IANCs during the study period. Another possibility is the increased use of antiviral therapy (oseltamivir) in all age groups in our cohort over the years during the study period. Beside symptomatic treatment to maintain hydration, control fever and nasal congestion, oseltamivir would be recommended to patients with confirmed influenza A or B infection if the presentation was within 2 days of onset of symptoms or when severe influenza-related complications developed. All doses were given twice daily for 5 days. Possible side effects are explained to the parents. The antiviral drug would only be started with parent's consent. It is known that antiviral therapy usage is associated with the reduction of complications (42, 43).

## Limitations

This is a retrospective study that quantitatively analysed the medical data of all paediatric patients with confirmed influenza A or B infection admitted to the public hospitals in Hong Kong. The public hospitals account for 85% of all the in-patient beds in Hong Kong. Our study's data were therefore unable to estimate the paediatric IANC admissions in the private hospitals. As such, the incidence rate of IANCs in our study may have been slightly underestimated. However, our study gave a good representation of data for those patients with severe IANCs as they would usually be transferred from the private hospitals to the public hospital for possible PICU care and further management. In this study, we were not able to compare the seasonal influenza vaccination coverage rates between our patients with and without IANCs because individual influenza vaccination records are not available in the current CDARS system. We therefore could not show a direct link that the decrease in IANC rate is related to the increase in influenza vaccination. As such, further studies should be carried out to investigate the effect of vaccination on influenza admission and IANCs in Hong Kong. Lastly, the current patient privacy policy within the CDARS system search limited our access to the detailed clinical data of each individual patient. We acknowledged the limitation that the subgrouping of febrile seizures into simple and complex nature is not possible with the current data search in our cohort too.

## Conclusions

This is a large population-based study of IANCs in paediatric in-patients over a 5-year period from 2014 to 2018. The significant clinical burden of influenza A and B infections in paediatric patients is reflected by the high hospitalisation rates. Patients with IANCs shared a higher clinical burden and had a higher mortality rate than non-IANC patients. Children <6 years old showed the highest estimated incidence of IANCs and death among paediatric patients with influenza A and B infection. Increased seasonal influenza vaccination coverage to this younger age group is therefore vital.

## Data Availability Statement

The original contributions presented in the study are included in the article/Supplementary Material, further inquiries can be directed to the corresponding author/s.

## Ethics Statement

The studies involving human participants were reviewed and approved by Institutional Review Board of the University of Hong Kong. Written informed consent from the participants' legal guardian/next of kin was not required to participate in this study in accordance with the national legislation and the institutional requirements.

## Author Contributions

MY and CL performed the analysis and wrote the first draft of the manuscript. WW, AH, and AC contributed to the investigation of influenza associated neurological complications. WW and HZ contributed to the methodology of the study. GC and SC contributed to conception and design of the study. All authors contributed to manuscript revision and approved the submitted version.

## Funding

This current study was supported by donation funding on Diagnosis and therapy development of neurological diseases and neuromuscular diseases (20009121) received by SC.

## Conflict of Interest

The authors declare that the research was conducted in the absence of any commercial or financial relationships that could be construed as a potential conflict of interest.

## Publisher's Note

All claims expressed in this article are solely those of the authors and do not necessarily represent those of their affiliated organizations, or those of the publisher, the editors and the reviewers. Any product that may be evaluated in this article, or claim that may be made by its manufacturer, is not guaranteed or endorsed by the publisher.

## Supplementary Material

Supplementary Figure 1 Composition of IANCs by influenza subtypes and age groups. **(A)** Composition of the different subtypes of IANCs in influenza A and B infections. **(B)**. Composition of the different age groups of IANCs in influenza A and B infections. Chi-square test was used to compare the percentages of IANCs in different influenza subtypes and age groups (Figures 2A,B). *p* < 0.05 is statistically significant.

Supplementary Figure 2 Data from the Department of Health - the seasonal influenza vaccination (SIV) coverage rate for HK children aged 6 months to <12 years from 2014/2015 to 2018/2019. The SIV coverage rate aged 6 months to <6 years increased from 18% in 2014/2015 to 35% in 2018/2019. The SIV coverage rate for children aged 6 months to <12 years increased from 18% in 2016/2017 to 45% in 2018/2019.

Supplementary Table 1 List of International Classification of Diseases (ICD) codes corresponding to influenza-associated neurological complications.

Supplementary Table 2 List of International Classification of Diseases (ICD) codes corresponding to pre-existing neurological diseases and coinfection with other virus/bacteria.

Supplementary Table 3 Hong Kong Resident Paediatric Population (<18 years old) data provided by the Census and Statistics Department, Hong Kong Special Administrative Region (HKSAR).

Supplementary Table 4 Incidence rate of IANCs per 100,000 population in different age groups.
