# Supplementary figures and images for: Clinical Spectrum and Burden of Influenza-Associated Neurological Complications in Hospitalised Paediatric Patients
Source: Front Pediatr. 2022 Jan 20;9:752816. doi: 10.3389/fped.2021.752816 (PMC8811455; doi:10.3389/fped.2021.752816)

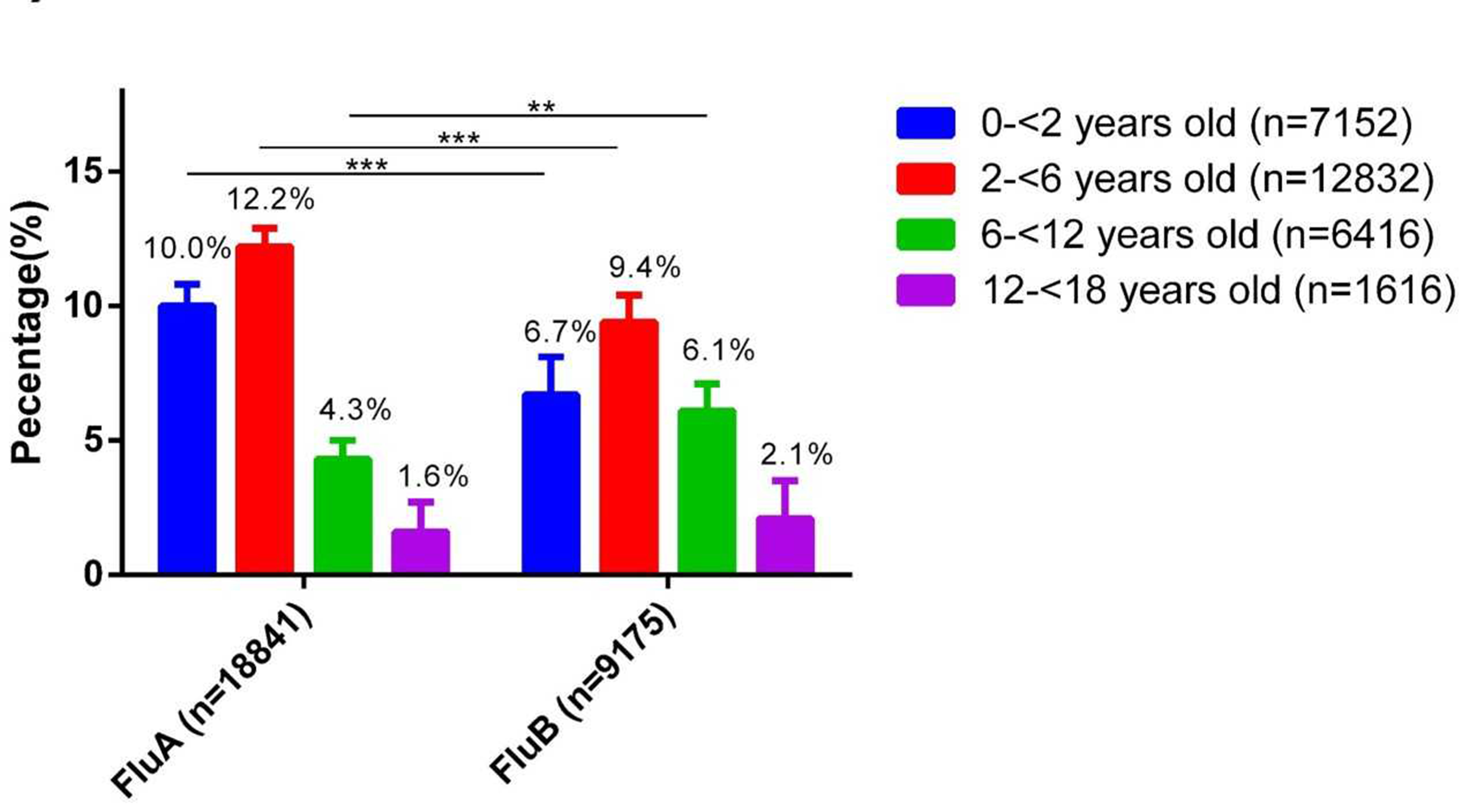

Supplement: Supplementary Figure 2 — Data from the Department of Health - the seasonal influenza vaccination (SIV) coverage rate for HK children aged 6 months to <12 years from 2014/2015 to 2018/2019. The SIV coverage rate aged 6 months to <6 years increased from 18% in 2014/2015 to 35% in 2018/2019. The SIV coverage rate for children aged 6 months to <12 years increased from 18% in 2016/2017 to 45% in 2018/2019. [file Image_2.JPG]
