# Supplementary material for: Clinical Spectrum and Burden of Influenza-Associated Neurological Complications in Hospitalised Paediatric Patients
Source: Front Pediatr. 2022 Jan 20;9:752816. doi: 10.3389/fped.2021.752816 (PMC8811455; doi:10.3389/fped.2021.752816)
Supplement: Supplementary Table 1 — List of International Classification of Diseases (ICD) codes corresponding to influenza-associated neurological complications. [file Table_1.docx]

**Supplementary Table 1: List of International Classification of Diseases (ICD) codes corresponding to influenza-associated neurological complications**

| Disease | ICD-9 code |
| --- | --- |
| Influenza A | 487.1 (3),487.1 (7),487.1 (9), 487.1 (11) |
| Influenza B | 487.1 (4) |
| Febrile Seizure (Febrile convulsions) /Seizure with Fever | 780.31 |
| Reye's Syndrome | 331.81 |
| Encephalitis/Encephalopathy | 323.9,348.3 |
| Viral meningitis | 47.9 |
| Guillain-Barré syndrome | 357.0(3) |
| Acute disseminated encephalomyelitis | 323.6 |
| Myositis | 728.0, 729.1 |
