# Supplementary material for: Clinical Spectrum and Burden of Influenza-Associated Neurological Complications in Hospitalised Paediatric Patients
Source: Front Pediatr. 2022 Jan 20;9:752816. doi: 10.3389/fped.2021.752816 (PMC8811455; doi:10.3389/fped.2021.752816)
Supplement: Supplementary Table 2 — List of International Classification of Diseases (ICD) codes corresponding to pre-existing neurological diseases and coinfection with other virus/bacteria. [file Table_2.docx]

**Supplementary Table 2: List of International Classification of Diseases (ICD) codes corresponding to pre-existing neurological diseases and coinfection with other virus / bacteria**

| Disease | ICD-9 code |
| --- | --- |
| Neurological disorder |  |
| Autistic disorder | 299.00/299.01 |
| Attention deficit disorder with hyperactivity | 314.01 |
| Cerebral artery occlusion, unspecified with cerebral infarction | 434.91 |
| Coma | 780.01 |
| Developmental delay | 315 |
| Down's syndrome | 758.0 |
| Encephalitis myelitis and encephalomyelitis | 323 |
| Epilepsy and recurrent seizures | 345 |
| Meningitis | 322 |
| Sanfilippo syndrome | 277.5 |
| Unspecified intellectual disabilities | 319 |
| Wolf-Hirschhorn syndrome | 758.3 |
| Co-virus infection | |
| Adenoviruse | 79.0(0) |
| Human Metapneumovirus | 79.89(8) |
| Parainfluenza | 79.89(4) |
| Respiratory syncytial virus | 79.6(0) |
| Rhinoviruse | 79.3(0) |
| Rotavirus | 8.61 |
| Co-bacterial infection |  |
| Campylobacter jejuni | 8.43 |
| Haemophilus influenzae | 41.50 |
| Legionella pneumophila | 482.83(1) |
| Mycobacteria | 31.9(0) |
| Meningococci | 36.9 |
| Mycoplasma | 41.81 |
