# Supplementary material for: Clinical Spectrum and Burden of Influenza-Associated Neurological Complications in Hospitalised Paediatric Patients
Source: Front Pediatr. 2022 Jan 20;9:752816. doi: 10.3389/fped.2021.752816 (PMC8811455; doi:10.3389/fped.2021.752816)
Supplement: Supplementary Table 3 — Hong Kong Resident Paediatric Population (<18 years old) data provided by the Census and Statistics Department, Hong Kong Special Administrative Region (HKSAR). [file Table_3.docx]

**Supplementary Table 3: Hong Kong Resident Paediatric Population (<18 years old) data provided by the Census and Statistics Department, Hong Kong Special Administrative Region (HKSAR)**

| Age group / Year | 2014 | 2015 | 2016 | 2017 | 2018 | 2014-2018 |
| --- | --- | --- | --- | --- | --- | --- |
| 0y to <2y | 94700 | 107100 | 110000 | 108800 | 105100 | 525700 |
| 2y to <6y | 228200 | 234300 | 234900 | 231900 | 229900 | 1159200 |
| 6y to <12y | 305600 | 317200 | 329900 | 348900 | 367100 | 1668700 |
| 12y to <18y | 386300 | 361400 | 341000 | 330300 | 327200 | 1746200 |
| 0y to <18y | 1014800 | 1020000 | 1015800 | 1019900 | 1029300 | 5099800 |
