# Supplementary material for: Clinical Spectrum and Burden of Influenza-Associated Neurological Complications in Hospitalised Paediatric Patients
Source: Front Pediatr. 2022 Jan 20;9:752816. doi: 10.3389/fped.2021.752816 (PMC8811455; doi:10.3389/fped.2021.752816)
Supplement: Supplementary Table 4 — Incidence rate of IANCs per 100,000 population in different age groups. [file Table_4.docx]

**Supplementary Table 4: Incidence rate of IANCs per 100,000 population in different age groups**

The data is presented as incidence rate per 100,000 population (95% confidence interval). The population-based incidence rate of IANCs was determined by the proportion of IANC cases admitted to public hospitals over the paediatric population of Hong Kong, as obtained from the Census and Statistics Department, Hong Kong Special Administrative Region. The incidence rate of hospitalisation was weighted by 0.85 to reflect the proportion of patients served by public hospitals in Hong Kong.

| Year | 2014 | | 2015 | | 2016 | | 2017 | | 2018 | | **2014-2018** | | |
| --- | --- | --- | --- | --- | --- | --- | --- | --- | --- | --- | --- | --- | --- |
| Types of Influenza | A | B | A | B | A | B | A | B | A | B | **A** | **B** | **A+B** |
| Age 0-<2y  Incidence rate  (95% CI) | 130  (108-158) | 24  (15-37) | 115  (95-140) | 8  (4-16) | 141  (119-167) | 31  (22-45) | 160  (136-188) | 9  (4-17) | 78  (62-99) | 47  (35-64) | **125**  **(102-148)** | **24**  **(14-34)** | **149**  **(123-174)** |
| Age 2-<6y  Incidence rate  (95% CI) | 107  (94-123) | 36  (28-45) | 98  (85-113) | 15  (10-21) | 128  (113-145) | 55  (45-66) | 142  (126-160) | 21  (15-28) | 76  (65-90) | 62  (52-74) | **110**  **(96-125)** | **37**  **(29-46)** | **148**  **(131-165)** |
| Age 6-<12y  Incidence rate  (95% CI) | 6  (4-10) | 13  (10-19) | 11  (8-16) | 6  (4-10) | 12  (8-17) | 17  (13-23) | 11  (8-15) | 8  (5-12) | 12  (9-16) | 19  (15-24) | **10**  **(7-14)** | **13**  **(9-17)** | **23**  **(18-29)** |
| Age 12-<18y  Incidence rate  (95% CI) | 1  (0-3) | 1  (0-3) | 1  (0-3) | 0 | 1  (0-3) | 1  (1-4) | 2  (1-4) | 1  (1-4) | 1  (0-3) | 1  (1-4) | **1**  **(0-2)** | **1**  **(0-2)** | **2**  **(0-4)** |
| Age 0-<18y  Incidence rate  (95% CI) | **39**  **(35-43)** | **15**  **(12-17)** | **38**  **(36-43)** | **6**  **(5-8)** | **49**  **(45-54)** | **22**  **(19-25)** | **53**  **(49-59)** | **9**  **(7-11)** | **29**  **(26-33)** | **26**  **23-29)** | **42**  **(38-46)** | **15**  **(13-18)** | **57**  **(52-62)** |
